# Photoredox chemistry in the synthesis of 2-aminoazoles implicated in prebiotic nucleic acid synthesis†
†Electronic supplementary information (ESI) available. CCDC 2024098. For ESI and crystallographic data in CIF or other electronic format see DOI: 10.1039/d0cc05752e


**DOI:** 10.1039/d0cc05752e

**Published:** 2020-10-08

**Authors:** Ziwei Liu, Long-Fei Wu, Andrew D. Bond, John D. Sutherland

**Affiliations:** a MRC Laboratory of Molecular Biology , Cambridge Biomedical Campus , Cambridge , UK . Email: johns@mrc-lmb.cam.ac.uk; b Department of Chemistry , University of Cambridge , Cambridge , UK

## Abstract

Prebiotically plausible ferrocyanide–ferricyanide photoredox cycling oxidatively converts thiourea to cyanamide, whilst HCN is reductively homologated to intermediates which either react directly with the cyanamide giving 2-aminoazoles, or have the potential to do so upon loss of HCN from the system. Thiourea itself is produced by heating ammonium thiocyanate, a product of the reaction of HCN and hydrogen sulfide under UV irradiation.

## Abstract 2

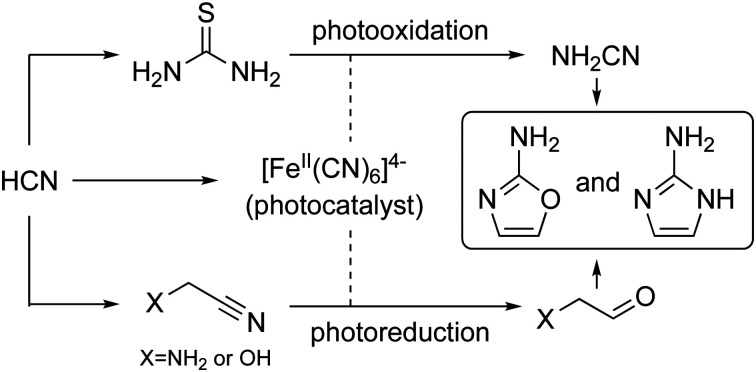
A direct link from cyanamide to cyanosulfidic chemistry *via* thiourea was demonstrated. 2-Aminoazoles were generated by photoredox cycling under prebiotically plausible conditions.

## 

Cyanamide **1** is pivotal to the synthesis of both 2-aminooxazole **2** (2-AO) and 2-aminoimidazole **3** (2-AI) through condensation with glycolaldehyde **4**. 2-AO **2** is formed in the absence of added ammonium salts and a mixture of **2** and 2-AI **3** is formed when such salts are added.^1^,^2^ Nitrile photoredox chemistry was previously reported to generate simple sugars, including glycolaldehyde **4** and glyceraldehyde.^3^–^6^ 2-AO **2** reacts with glyceraldehyde to generate predominantly *arabino*- and *ribo*-configured pentose aminooxazolines, the former of which is a precursor of pyrimidine ribonucleotides^1^,^2^ and the latter of which is a precursor of both pyrimidine ribonucleosides^7^ and purine deoxyribonucleosides.^8^,^9^ 5′-Phosphoro-2-aminoimidazolides derived from 2-AI **3** and transiently activated nucleotides participate in non-enzymatic RNA replication chemistry *via* the intermediacy of 5′-5′-imidazolium-bridged dinucleotides.^10^,^11^

Despite the importance of cyanamide **1** in potential prebiotic chemistry there have been few attempts at investigating its provenance on early Earth. An attempt to synthesize **1** by subjecting mixtures of cyanide and ammonia to UV irradiation gave low yields of dicyanamide from which the intermediacy of cyanamide **1** was inferred, but no **1** was detected.^12^ Recently the synthesis of cyanamide **1** in low yield by carefully dosed radiolysis of a solution containing hydrogen cyanide and high concentrations of chloride (5.1 M) and ammonium (0.1 M) ions was reported.^13^ It has also been proposed that calcium cyanamide generated by thermal decomposition of calcium ferrocyanide (Ca_2_[Fe^II^(CN)_6_]) could have been a source of cyanamide **1** through salt hydrolysis.^14^ However, the formation of Ca_2_[Fe^II^(CN)_6_] on early Earth has been challenged recently,^15^ modelling suggesting that under a CO_2_-rich atmosphere, calcium ions would precipitate as the carbonate leaving ferrocyanide to precipitate as its sodium salt. Thus, unless CO_2_ was transiently removed from Earth's atmosphere by reduction following collision with an iron- and nickel-rich impactor, for example, the presence of calcium cyanamide seems unlikely.

Cyanamide **1** has been reported as a product of the decomposition of the thiourea oxidation product, formamidine disulfide under non-acidic conditions.^16^,^17^ We reasoned that thiourea **5** might be oxidized by ferricyanide [Fe^III^(CN)_6_]^3–^ in a photoredox cycle also involving ferrocyanide.^5^,^6^ Here we show that cyanamide **1** can be generated by irradiating thiourea **5** in the presence of a catalytic amount of ferrocyanide in aqueous solution through photoredox coupling to cyanide reduction. We further show how this can lead to the generation of 2-AO **2** and 2-AI **3**. Lastly, we outline a synthesis and purification of thiourea **5** including a plausible prebiotic crystallization step.^18^

A mixture of ^13^C-labelled thiourea **5** (20 mM) with potassium ferricyanide (40 mM) in phosphate buffer (pH = 8, 500 mM) was incubated at 23 °C. After 3 days, cyanamide **1** was detected as the major product by quantitative ^13^C-NMR spectroscopy (45% yield, Fig. 1). Meanwhile, another mixture of cyanamide **1** (50 mM), glycolaldehyde **4** (50 mM) in phosphate buffer (pH = 8, 200 mM) was incubated at 23 °C for 19 hours. 2-AO **2** and its hydrate **6** were observed in yields of 29% and 65%, respectively (Fig. S1, ESI†). Thus, conditions for the synthesis of cyanamide **1** from thiourea **5** are consistent with those required for the reaction of **1** with glycolaldehyde **4** although the majority of the product of the latter reaction is in the form of its hydrate.

**Fig. 1:**
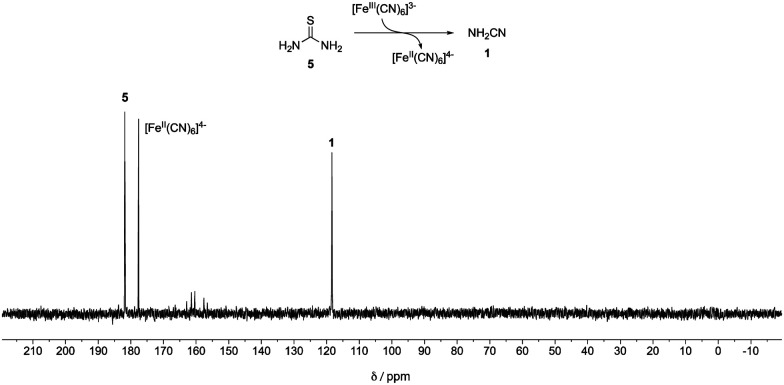
^13^C-NMR Spectra of a mixture of ^13^C-labelled thiourea **5** (20 mM) and potassium ferricyanide (40 mM) in 500 mM phosphate buffer (pH = 8, in 10% D_2_O in H_2_O) after 3 days.

Previously, we reported that hydrated electrons produced by the known photoionization of ferrocyanide [Fe^II^(CN)_6_]^4–^ drive the reductive homologation of hydrogen cyanide **7** (HCN) to the simple carbohydrates, glycolaldehyde **4** and glyceraldehyde in a Kiliani-Fischer-type process.^5^ In this earlier work, the ferricyanide resulting from the photoionization of ferrocyanide was reduced back to the latter with bisulfite enabling photoredox cycling. We now wondered if such ferrocyanide–ferricyanide photoredox cycling could couple the oxidation of thiourea **5** with the reduction of glycolonitrile **8**, an intermediate in the Kiliani–Fischer-type process (Scheme 1, stage 2). This would generate cyanamide **1** and glycolaldehyde **4** simultaneously, potentially allowing subsequent formation of 2-AO **2***in situ*. Were this to work, it might then be possible to couple the oxidation of thiourea **5** with the multi-step reductive homologation of HCN **7** to glycolaldehyde **4**.

**Scheme 1:**
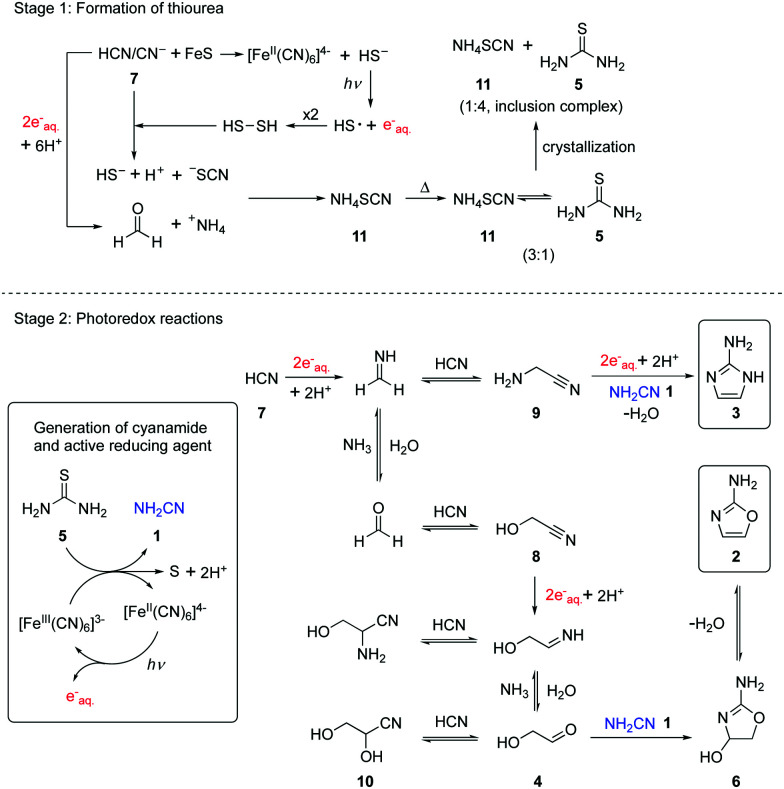
Schematic representation of the systems chemistry network producing thiourea **5**, reductive homologation of HCN **7**, and the formation of 2-AO **2** and 2-AI **3** by photoredox chemistry.

Because ferrocyanide and ferricyanide undergo photoaquation (in addition to the photoionization of ferrocyanide) at the 254 nm wavelength we employ in our exploratory prebiotic photochemistry experiments^19^ and the photoaquation products interfere with NMR analysis, cyanide was added to increase the rate of the back reactions which reverse photoaquation and thus shift photostationary equilibria in favour of fully cyanated complexes. We realized that this addition would mean that we would not be able to say whether any 2-AO **2** resulted from the direct reduction of glycolonitrile **8**, or the reductive homologation of HCN **7**, but we reasoned that by having **8** present in addition to **7**, we would maximize our chances of producing and detecting 2-AO **2**. Accordingly, a mixture of glycolonitrile **8** (50 mM), ^13^C-labelled thiourea **5** (50 mM), potassium ferrocyanide (5 mM), and potassium cyanide (30 mM) in phosphate buffer (pH = 8, 200 mM) was subjected to UV irradiation. After 7 hours irradiation, 2-AO **2** was observed in 4% yield by ^1^H-NMR spectroscopy, which suggests that the transformation of thiourea **5** to cyanamide **1** is coupled to the synthesis of glycolaldehyde **4** in this photoredox system. Other HCN reductive homologation products, aminoacetonitrile **9** (8% yield) and glyceronitrile **10** (27% yield) were observed as well (Fig. S2 and Table S1, ESI,† Scheme 1). The formation of aminoacetonitrile **9** indicated that **7** was reduced to methanimine, at least some of which was trapped by addition of cyanide rather than undergoing hydrolysis to formaldehyde, trapping of which by cyanide addition, generates glycolonitrile **8**.^3^,^4^ The formation of glyceronitrile **10** in 27% yield suggested that cyanide was outcompeting cyanamide **1** in reaction with glycolaldehyde **4**. As cyanohydrin formation is reversible, this did not overly concern us at this point, indeed we could see a benefit to sequestering glycolaldehyde **4** as the cyanohydrin **10**, thereby delaying the synthesis of 2-AO **2** from **4** and cyanamide **1**, because of the reported photolability of 2-AO **2**.^20^ We thus investigated whether the precursors to 2-AO **2** could be produced from thiourea **5** and HCN **7** directly. A mixture of ^13^C-labelled thiourea **5** (50 mM), potassium ferrocyanide (5 mM) and potassium cyanide (100 mM) in phosphate buffer (pH = 7 or 8, 200 mM) was irradiated for 6 hours. Glycolonitrile **8** (24% yield at pH 7 and 16% yield at pH 8), aminoacetonitrile **9** (28% yield at pH 7 and 24% yield at pH 8), glyceronitrile **10** (12% yield at pH 7 and 11% yield at pH 8), and 2-AI **3** (4% at both pH values) were observed by ^1^H-NMR spectroscopy, and ^13^C-labelled cyanamide **1** was observed by ^13^C-NMR spectroscopy (Fig. S3–S5 and Table S1, ESI†). 2-AO **2** was not observed directly, but the liberation of glycolaldehyde **4** from glyceronitrile **10** thereby allowing reaction with cyanamide **1** giving 2-AO **2** (and its hydrate **6**) by wet-dry cycling was reported recently.^13^ Thus, what has been termed a continuous reaction network^13^ from HCN **7** and thiourea **5** is possible through photoredox cycling. However, we note that further progression from 2-AO **2** to ribo- and deoxyribonucleosides almost certainly requires discontinuity in the synthesis and we have shown that such discontinuities can be accomplished by flow chemistry which mimics a fluvial scenario on early Earth.^6^ Furthermore, exploitation of the conglomerate crystallization of a later intermediate in the nucleoside synthesis is most easily achieved by flow as it allows plausible separation of crystalline material from mother liquor. Such crystallization removes by-products, salts and excess reagents and effectively resets the dial regarding yield in the sense that low yields mainly impact multistep prebiotic synthesis in a negative way because of associated lowering of product purity.

We considered it noteworthy that 2-AI **3** observed in our experiments (Fig. S3 and S4. ESI†) is an essential compound in the nonenzymatic copying of oligoribonucleotides as reported by Szostak and co-workers.^10^ To further investigate the route by which 2-AI **3** is produced by photoredox chemistry, a mixture of aminoacetonitrile **9** (50 mM), ^13^C-labelled thiourea **5** (50 mM), potassium ferrocyanide (5 mM) and potassium cyanide (30 mM) in phosphate buffer (pH = 7 or 8, 200 mM) was irradiated for 14 hours. 2-^13^C-2-AI **3** (50% yield at pH 7 and 20% yield at pH 8) was observed by ^1^H-NMR spectroscopy (Fig. S6 and Table S1, ESI†). This indicates that in our system, 2-AI **3** is formed from the reduction of aminoacetonitrile **9** to the imine of aminoacetaldehyde followed by addition of cyanamide **1**. Thus, a synthesis of 2-AI **3** that does not require high concentrations of ammonium ions has been uncovered.

The foregoing results show that thiourea **5** is an effective precursor of cyanamide **1** through ferrocyanide–ferricyanide photoredox cycling and so it is reasonable to ask how thiourea **5** could have been produced prebiotically on early Earth. According to the cyanosulfidic chemistry scenario we have previously described,^14^,^21^ it is conceivable that a buffered cyanide solution corroded metal sulfides (including traces of copper sulfide) releasing hydrosulfide (HS^–^) into solution.^22^,^23^ Irradiation of such a mixture results in photoredox cycling with reduction of HCN **7** and oxidation of HS^–^, but instead of products with S–S bonds accumulating, cyanide cleaves them as they are formed generating thiocyanate. Meanwhile, hydrolysis of imines resulting from nitrile reduction in the reductive homologation of HCN **7** generates ammonium ions as counter ions to the thiocyanate. Absent cyanometallate photoredox cycling, the irradiation of buffered solutions containing HCN **7** and HS^–^ still results in reductive homologation, albeit more slowly. In this case, photoionization of HS^–^ generates hydrated electrons and thiocyanate is ultimately produced from the resultant HS˙/^–^S˙ radicals and HCN **7** (either *via* the transient formation of S–S bonds and cleavage thereof by cyanide, or by addition of HS˙/^–^S˙ to HCN **7** followed by hydrogen atom abstraction by a second HS˙/^–^S˙). To assess thiocyanate production in a cyanometallate-free system, a solution containing cyanide (50 mM) and hydrosulfide (50 mM) at pH = 8 was irradiated with 254 nm light for 1 hour to give thiocyanate (70% yield) as the major product according to quantitative ^13^C-NMR spectroscopy (Fig. S7, ESI†). Previously, we focused on the homologation products of HCN **7** in the various systems we have investigated and regarded the ammonium thiocyanate **11** as waste, but a central tenet of synthetic systems chemistry is that there is ideally no waste and so we surveyed the literature for reactions of **11**. More than 150 years ago in a classic study on par with Wöhler's synthesis of urea, it was reported that heating ammonium thiocyanate **11** in the dry state at 170 °C gave a 3 : 1 equilibrium mixture of ammonium thiocyanate **11** and thiourea **5**.^24^ The simplicity of this procedure appealed to us because it would correspond to a geochemical scenario in which a body of water that contained ammonium thiocyanate **11** was geothermally heated to dryness at first and then beyond. In this early study it was further reported that if the 3 : 1 mixture of ammonium thiocyanate **11** and thiourea **5** is dissolved in water with heating and cooled, fine, long, silky crystals are deposited which upon recrystallization give thiourea. A follow-on study reported that the first formed fine, long, silky crystals are co-crystals of ammonium thiocyanate **11** and thiourea **5** now in a 1 : 3 ratio.^25^ We repeated the crystallization process to further probe this, suspecting that these earlier authors had obtained crystals of a thiourea inclusion complex, species first characterized as such in the mid 1940's.^26^ When a 3 : 1 mixture of ammonium thiocyanate **11** and thiourea **5** in aqueous solution was subject to crystallization conditions, white needles were obtained. X-ray crystallographic analysis showed them to be of an inclusion complex with a 1 : 4 ratio of ammonium thiocyanate **11** and thiourea **5** (Fig. S8, ESI†).^27^ If this 1 : 4 ratio inclusion complex was recrystallized from water, hexagonal crystals, of pure thiourea **5**, were formed.

As the inclusion complex requires only one crystallization to form, a 1 : 4 mixture of ammonium thiocyanate **11** and thiourea **5** seems more prebiotically plausible than pure thiourea **5**. Given that thiocyanate can undergo photoionization, or photodissociation (to cyanide anion and ^3^P_*J*_ S atoms) upon irradiation,^28^,^29^ we studied ferrocyanide–ferricyanide photoredox chemistry in the presence of ammonium thiocyanate **11**. A solution containing ammonium thiocyanate **11** (20 mM), thiourea **5** (80 mM), KCN (100 mM), potassium ferrocyanide (5 mM) in phosphate buffer (pH = 6, 200 mM) was irradiated with 254 nm light. After 19 hours, glycolonitrile **8** (36%), aminoacetonitrile **9** (13%), glyceronitrile **10** (47%) and 2-AI **3** (4%) were observed by ^1^H-NMR spectroscopy (Fig. S9, ESI†). The results indicated that ammonium thiocyanate **11** did not adversely affect the ferrocyanide–ferricyanide photoredox system.

In summary, a direct link of cyanamide **1** to cyanosulfidic chemistry *via* thiourea **5** was developed by geologically plausible crystallization (Scheme 1). Furthermore, a catalytic photoredox cycling system was established which starts from thiourea **5** and HCN **7** or glycolonitrile **8**. Homologation products of **7**/reduction products of **8** react with *in situ* generated cyanamide **1** to form 2-aminoimidazole **3** and 2-aminooxazole **2**. These findings reinforce the cyanosulfidic scenario in which HCN **7** is central to the synthesis of protein, lipid, and nucleic acid building blocks.

## Conflicts of interest

There are no conflicts to declare.

## Supplementary Material

Supplementary information

Crystal structure data
