# Supplementary material for: Photoredox chemistry in the synthesis of 2-aminoazoles implicated in prebiotic nucleic acid synthesis
Source: Chem Commun (Camb). 2020 Oct 8;56(88):13563–6. doi: 10.1039/d0cc05752e (PMC7808312; doi:10.1039/d0cc05752e)
Supplement: Supplementary file 1 [file CC-056-D0CC05752E-s001.pdf]

Electronic Supplementary Material (ESI) for ChemComm.
This journal is © The Royal Society of Chemistry 2020

Supporting Information

Simultaneous photoredox chemical production of two 2-aminoazoles involved in prebiotic nucleic acid synthesis.

Ziwei Liu[a], Long-Fei Wu [a], Andrew D. Bond[b], and John D. Sutherland*[a]

Materials and methods. All reagents and deuterated solvents used for reactions and spiking experiments were purchased from Sigma-Aldrich or Santa Cruz Biotechnology and were used without further purification. All photochemical reactions were carried out in Norell Suprasil quartz NMR tubes purchased from Sigma-Aldrich using Hg lamps with principal emission at 254 nm in a Rayonet photochemical chamber reactor RPR-200, acquired from The Southern New England Ultraviolet Company. A Mettler Toledo SevenEasy pH Meter S20 was used to monitor the pH, and degassed H_2O or D_2O was achieved by four times of freeze-pump-thaw cycling. ^{1}H-, and ^{13}C-nuclear magnetic resonance (NMR) spectra were acquired using a Bruker Ultrashield 400 Plus or Bruker Ascend 400 operating at 400.1, and 100.6 MHz, respectively. Samples consisting of H_2O/D_2O mixtures were analyzed using HOD suppression to collect ^{1}H-NMR data. The quantitative ^{13}C-NMR spectra were acquired with inverse-gated decoupling, with a 90° excitation pulse and an inter-pulse delay of 70 seconds. ^{13}C longitudinal relaxation time constants (T1) were measured to be no greater than 13 seconds for any of the ^{13}C resonances at 100.6 MHz. Chemical shifts (δ) are shown in ppm. Coupling constants (*J*) are given in Hertz and the notations s, d, t represent the multiplicities singlet, doublet, and triplet signal. The conversion yields were determined by relative integrations of the signals using a known amount of acetamide as internal reference in the ^{1}H-NMR spectrum. X-ray diffraction data were collected on a Nonius KappaCCD instrument using graphite-monochromated MoKα radiation (λ = 0.7107 Å). The crystal was held at 180(2) K using an Oxford Cryosystems open-flow N_2 cryostat.

Synthesis of cyanamide 1 from thiourea 5 in dark. ^{13}C-labelled thiourea **5** (0.01 mmol) and sodium dihydrogen phosphate (0.25 mmol) were dissolved in H_2O/D_2O (9:1, 0.4 mL), and the pH was adjusted to the reported value with NaOH/HCl. In parallel, potassium ferricyanide (0.02 mmol) was dissolved in H_2O/D_2O (9:1, 0.1 mL). Then these two solutions were mixed.

The resultant solution was monitored by ^{13}C-NMR spectroscopy.

Synthesis of 2-AO 2 from cyanamide 1 and glycolaldehyde 4. Glycolaldehyde **4** (0.025 mmol) and sodium dihydrogen phosphate (0.1 mmol) were dissolved in H_2O/D_2O (9:1, 0.4 mL), and the pH was adjusted to 8 with NaOH/HCl. In parallel, cyanamide (0.025 mmol) was dissolved in H_2O/D_2O (9:1, 0.1 mL). Then these two solutions were mixed. The resultant solution was monitored by ^{1}H-NMR spectroscopy.

Photoredox synthesis of 2-AO 2 from glycolonitrile 8 and thiourea 5. Glycolonitrile **8** (0.025 mmol), ^{13}C-labelled thiourea **5** (0.025 mmol), potassium ferrocyanide (0.0025 mmol), potassium cyanide (0.015 mmol), and sodium dihydrogen phosphate (0.1 mmol) were dissolved in H_2O/D_2O (9:1, 0.5 mL). After the pH was adjusted to the reported value with NaOH/HCl, the mixture was transferred to a sealed quartz NMR tube and irradiated for a reported time. The resultant solution was analyzed by ^{1}H- or ^{13}C-NMR spectroscopy.

Photoredox synthesis of 2-AI 3 from KCN and thiourea 5. ^{13}C-labelled thiourea **5** (0.025 mmol), potassium ferrocyanide (0.0025 mmol), potassium cyanide (0.05 mmol), and sodium dihydrogen phosphate (0.1 mmol) were dissolved in H_2O/D_2O (9:1, 0.5 mL). After the pH was adjusted to the reported value with NaOH/HCl, the mixture was transferred to a sealed quartz NMR tube and irradiated for a reported time. The resultant solution was analyzed by ^{1}H- or ^{13}C-NMR spectroscopy.

Photoredox synthesis of 2-AI 3 from aminoacetonitrile 9 and thiourea 5. Aminoacetonitrile **9** (0.025 mmol), ^{13}C-labelled thiourea **5** (0.025 mmol), potassium ferrocyanide (0.0025 mmol), potassium cyanide (0.015 mmol), and sodium dihydrogen phosphate (0.1 mmol) were dissolved in H_2O/D_2O (9:1, 0.5 mL). After the pH was adjusted to the reported value with NaOH/HCl, the mixture was transferred to a sealed quartz NMR tube and irradiated for a reported time. The resultant solution was analyzed by ^{1}H- or ^{13}C-NMR spectroscopy.

Synthesis of thiocyanate by irradiating KCN and NaHS. ^{13}C-Labelled KCN (0.025 mmol), and NaHS (0.025 mmol) were dissolved in degassed H_2O/D_2O (9:1, 0.5 mL). After the pH was adjusted to the reported value with degassed NaOH/HCl, the mixture was transferred to a sealed quartz NMR tube and irradiated for a reported time. The resultant solution was analyzed by ^{13}C-NMR spectroscopy.

Recrystallization process. Ammonium thiocyanate **11** (30 mmol), and thiourea **5** (10 mmol)

were suspended in H_2O (3 mL). The suspension was heated until all solid dissolved, and allowed to stand at room temperature in an open round bottom flask until crystals formed. A single crystal was analyzed by X-ray crystallography.

Photoredox synthesis of 2-AI 3 from KCN and thiourea 5 with thiocyanate 11. ^{13}C-labelled thiourea **5** (0.04 mmol), ammonium thiocyanate **11** (0.01 mmol) potassium ferrocyanide (0.0025 mmol), potassium cyanide (0.05 mmol), and sodium dihydrogen phosphate (0.1 mmol) were dissolved in H_2O/D_2O (9:1, 0.5 mL). After the pH was adjusted to the reported value with NaOH/HCl, the mixture was transferred to a sealed quartz NMR tube and irradiated for a reported time. The resultant solution was analyzed by ^1H- or ^{13}C-NMR spectroscopy.

Synthesis of 2-AO hydrate 6. 2-AO **2** (0.01 mmol), and sodium dihydrogen phosphate (0.1 mmol) were dissolved in in H_2O/D_2O (9:1, 0.5 mL). After the pH was adjusted to 7 with NaOH/HCl, the resultant solution was allowed to stand at room temperature for 19 hours then analyzed by ^1H-NMR spectroscopy. 2-AO **2**: ^1H-NMR (400 MHz, D_2O) δ 7.21 (d, J = 1.1 Hz, 1H), 6.69 (d, J = 1.1 Hz, 1H). 2-AO hydrate **6**: ^1H-NMR (400 MHz, D_2O) δ 5.68 (dd, J = 6.1, 2.2 Hz, 1H), 4.44 (dd, J = 10.3, 2.2 Hz, 1H).

Synthesis of glyceronitrile 10. Glycolaldehyde **4** (0.05 mmol), and KCN (0.055 mmol) were dissolved in in H_2O/D_2O (9:1, 0.5 mL). After the pH was adjusted to 9 with NaOH/HCl, the resultant solution was analyzed by ^1H-NMR spectroscopy. ^1H-NMR (400 MHz, D_2O) δ 4.65 (t, J = 4.8 Hz, 1H), 3.76 (d, J = 4.8 Hz, 2H).

Synthesis of serine nitrile. Glycolaldehyde **4** (0.05 mmol), KCN (0.055 mmol), and ammonium chloride (0.25 mmol) were dissolved in in H_2O/D_2O (9:1, 0.5 mL). After the pH was adjusted to 9 with NaOH/HCl, the resultant solution was allowed to stand at room temperature for 65 hours then analyzed by ^1H-NMR spectroscopy.

Synthesis of formyl aminoacetonitrile. Aminoacetonitrile free base (1 mmol) and formic acid (1 mmol) were dissolved in dichloromethane. Then *N*,*N*'-dicyclohexylcarbodiimide (DCC, 2 mmol) was added to the solution. The mixture was stirred overnight at room temperature. After filtering off a precipitate, the residue was evaporated to dryness under vacuum, to give the product (95 % yield). ^1H-NMR (400 MHz, D_2O) δ 8.12 (s, 1H), 4.20 (s, 2H).

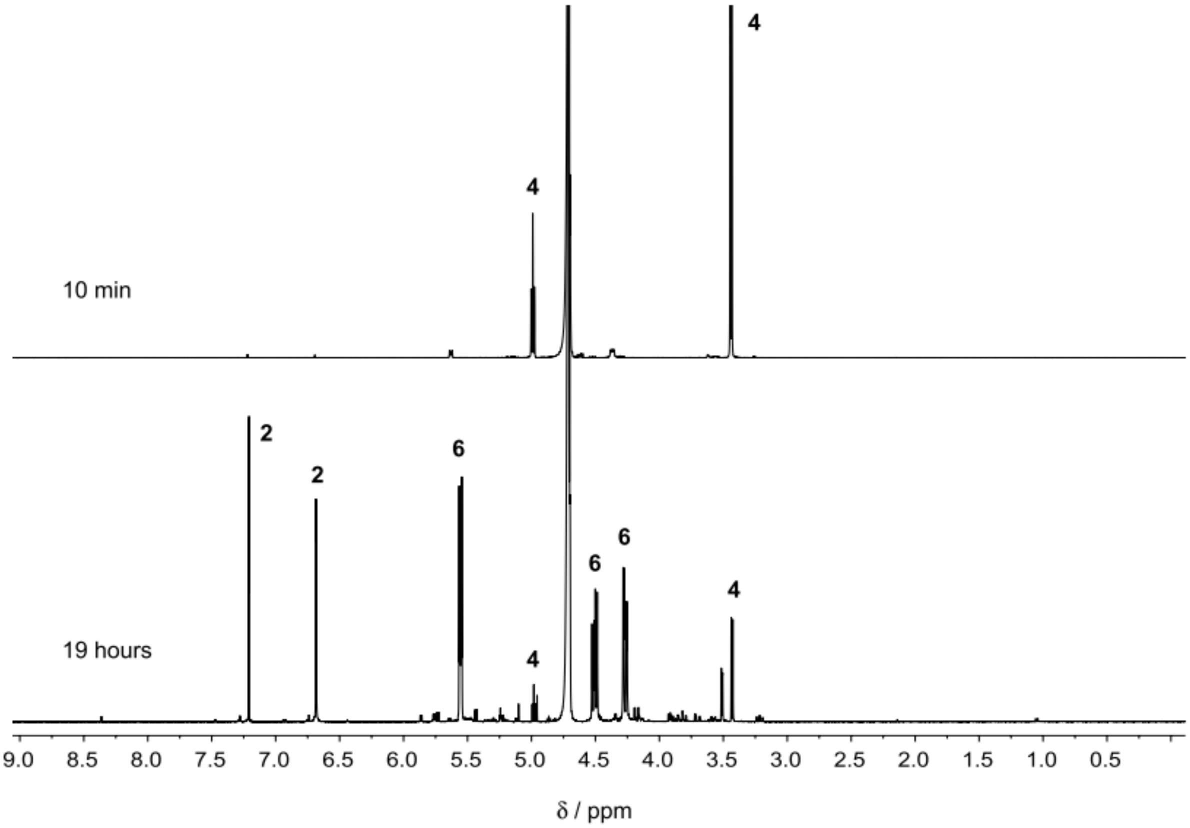

Fig. S1. ^{1}H-NMR Spectra of a mixture of glycolaldehyde **4** (50 mM), and cyanamide **1** (50 mM) in 200 mM phosphate buffer (pH = 8, in 10% D_2O in H_2O) after 10 min and 19 hours.

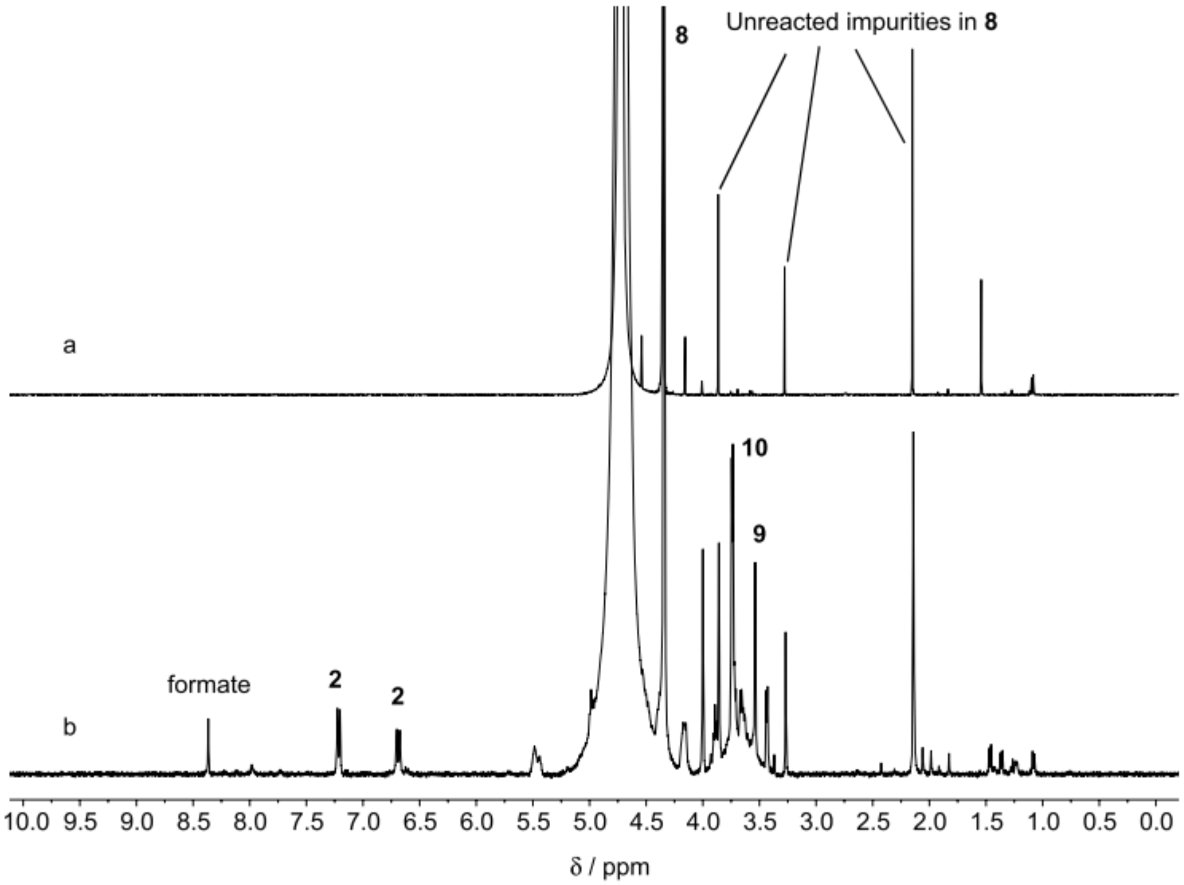

Fig. S2. ^{1}H-NMR Spectra of the reaction mixture with 50 mM glycolonitrile **8**, 50 mM ^{13}C-labelled thiourea **5**, 5 mM potassium ferrocyanide, and 30 mM KCN **7** in 200 mM phosphate buffer (pH = 8, in 10% D_2O in H_2O) (a) before irradiation; (b) irradiation at 254 nm for 7 hours.

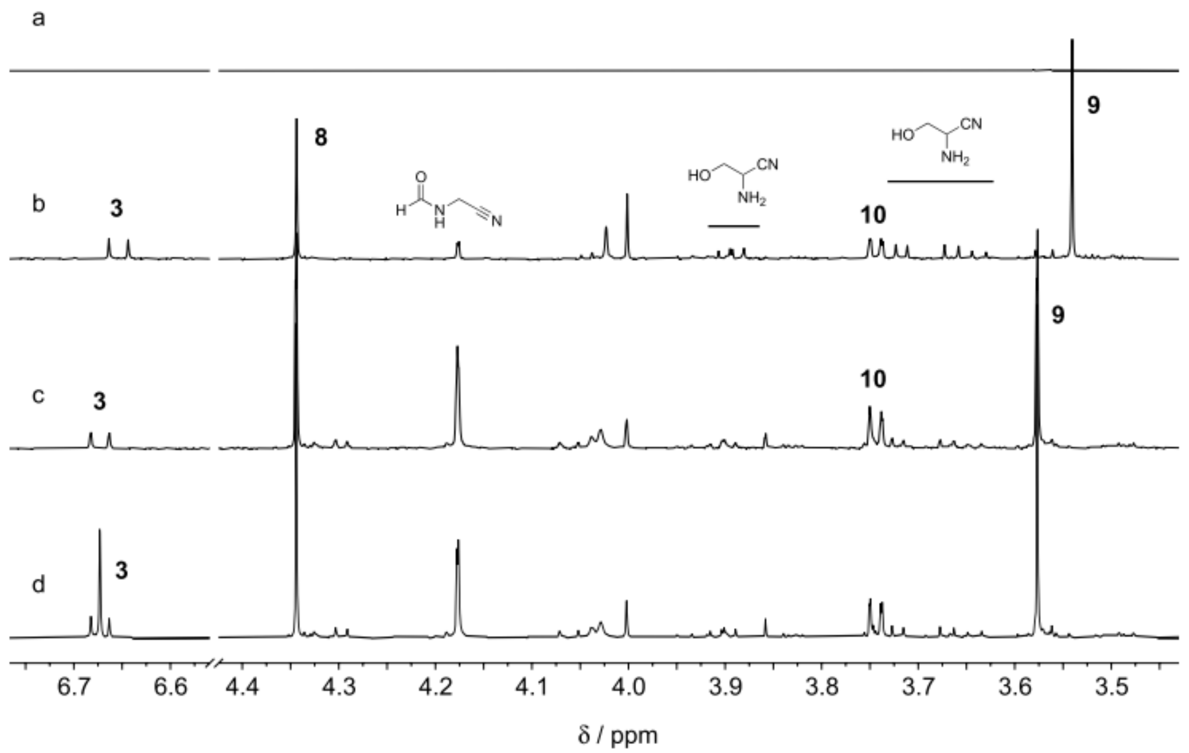

Fig. S3. ^{1}H-NMR Spectra of the reaction mixture with 50 mM ^{13}C-labelled thiourea **5**, 5 mM potassium ferrocyanide, and 100 mM KCN **7** in 200 mM phosphate buffer (pH =7 or 8, in 10% D_2O in H_2O) (a) before irradiation; (b) irradiation at 254 nm for 6 hours in a pH = 8 phosphate buffer; (c) irradiation at 254 nm for 6 hours in a pH = 7 phosphate buffer; (d) as (c) spiking with unlabeled authentic 2-AI **3**.

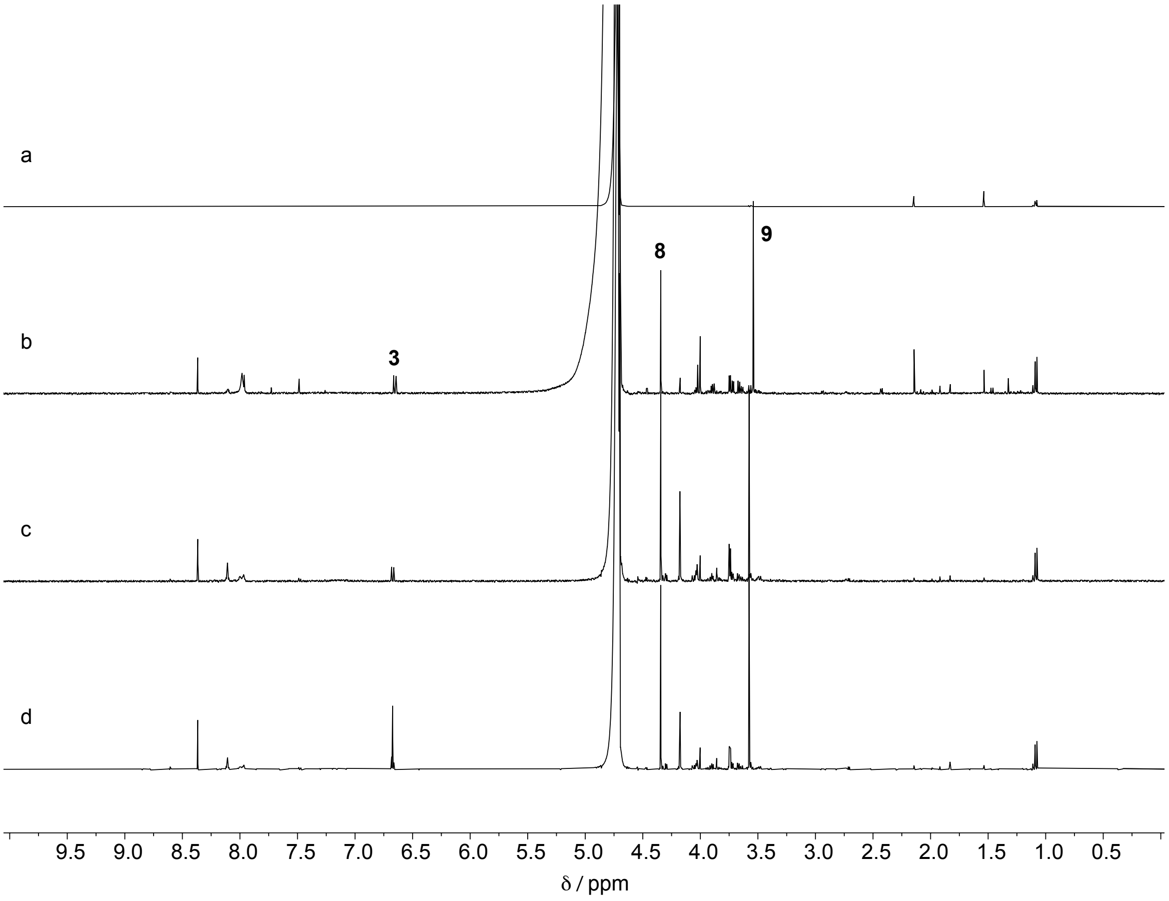

Fig. S4. Full spectra of Fig. S3.

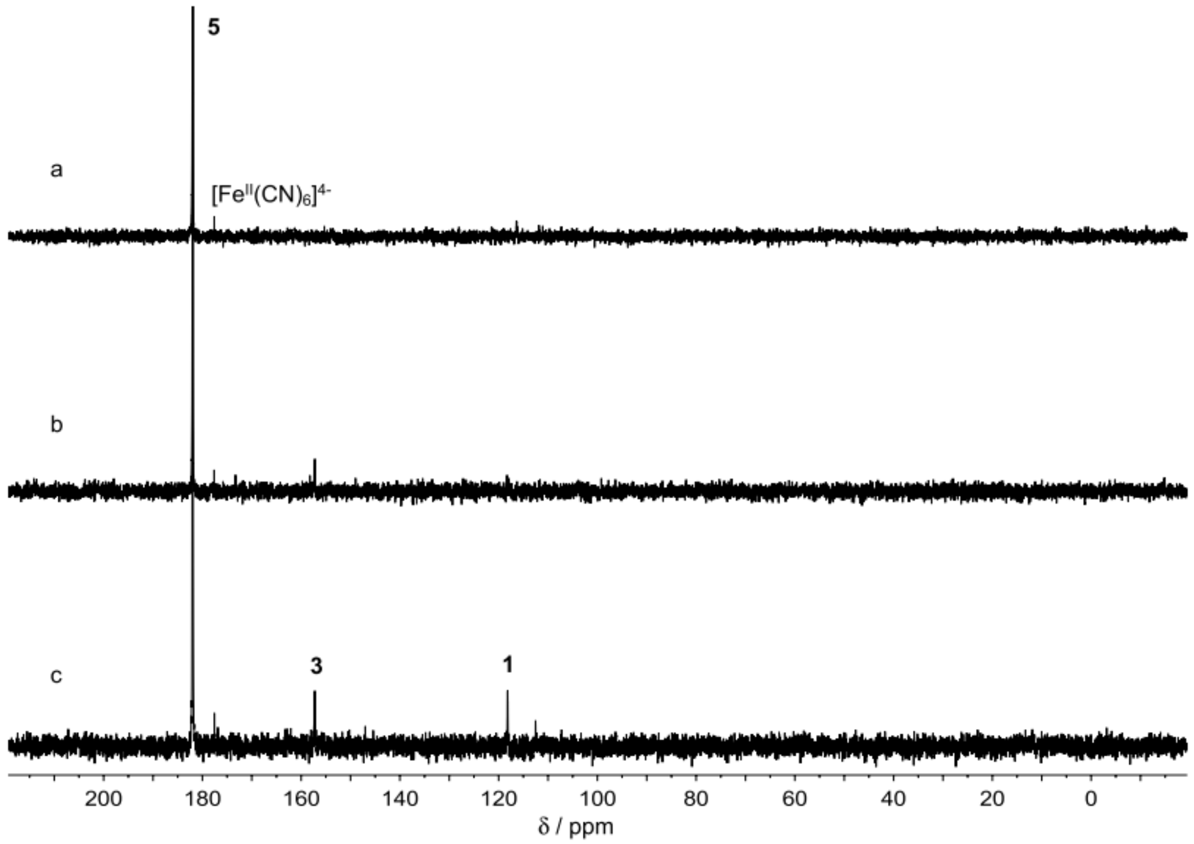

Fig. S5. ^{13}C-NMR Spectra of the reaction mixture with 50 mM ^{13}C-labelled thiourea **5**, 5 mM potassium ferrocyanide, and 100 mM KCN **7** in 200 mM phosphate buffer (pH =7 or 8, in 10% D_2O in H_2O) (a) before irradiation; (b) irradiation at 254 nm for 6 hours in a pH = 8 phosphate buffer; (c) irradiation at 254 nm for 6 hours in a pH = 7 phosphate buffer.

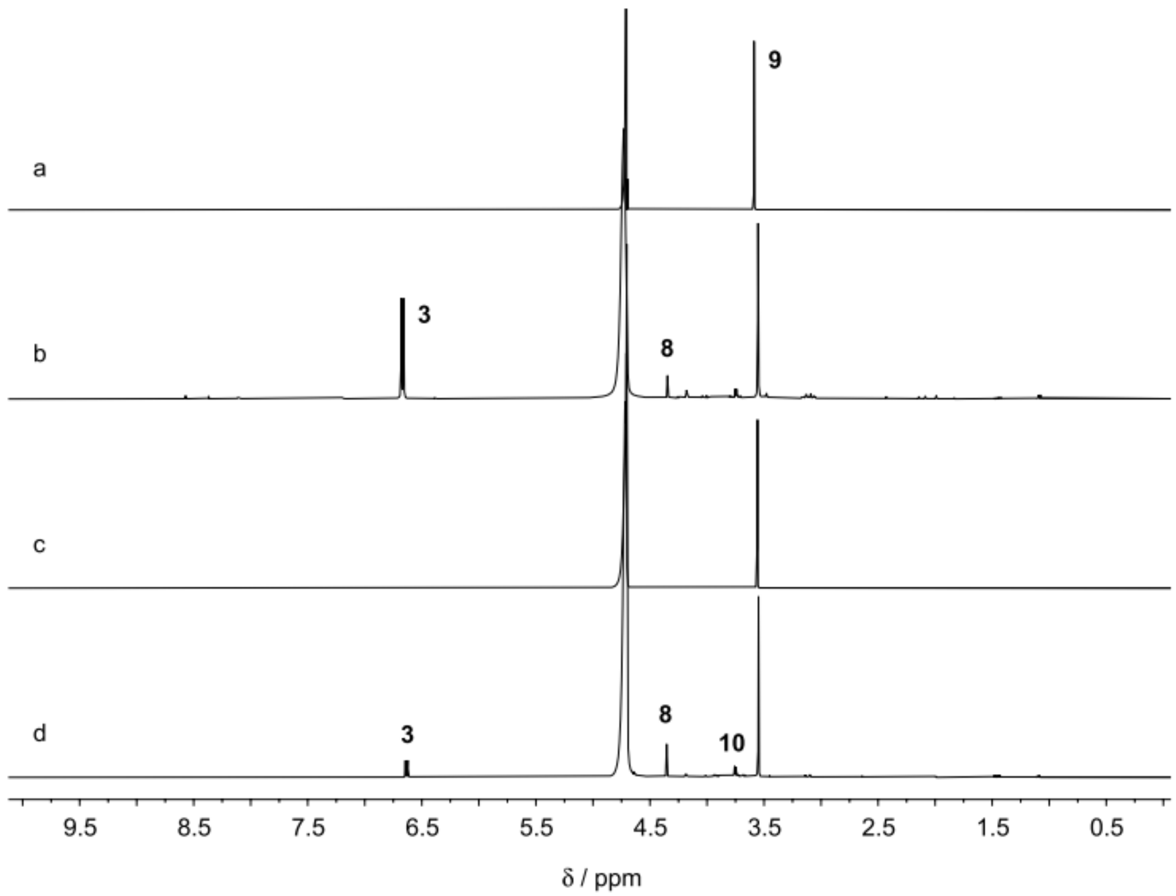

Fig. S6. ^{1}H-NMR Spectra of the reaction mixture with 50 mM aminoacetonitrile **9**, 50 mM ^{13}C-labelled thiourea **5**, 5 mM potassium ferrocyanide, and 30 mM KCN **7** in 200 mM phosphate buffer (pH = 7 or 8, in 10% D_2O in H_2O) (a) before irradiation in a pH = 7 buffer; (b) as (a) irradiation at 254 nm for 14 hours; (c) before irradiation in a pH = 8 buffer; (d) as (c) irradiation at 254 nm for 14 hours

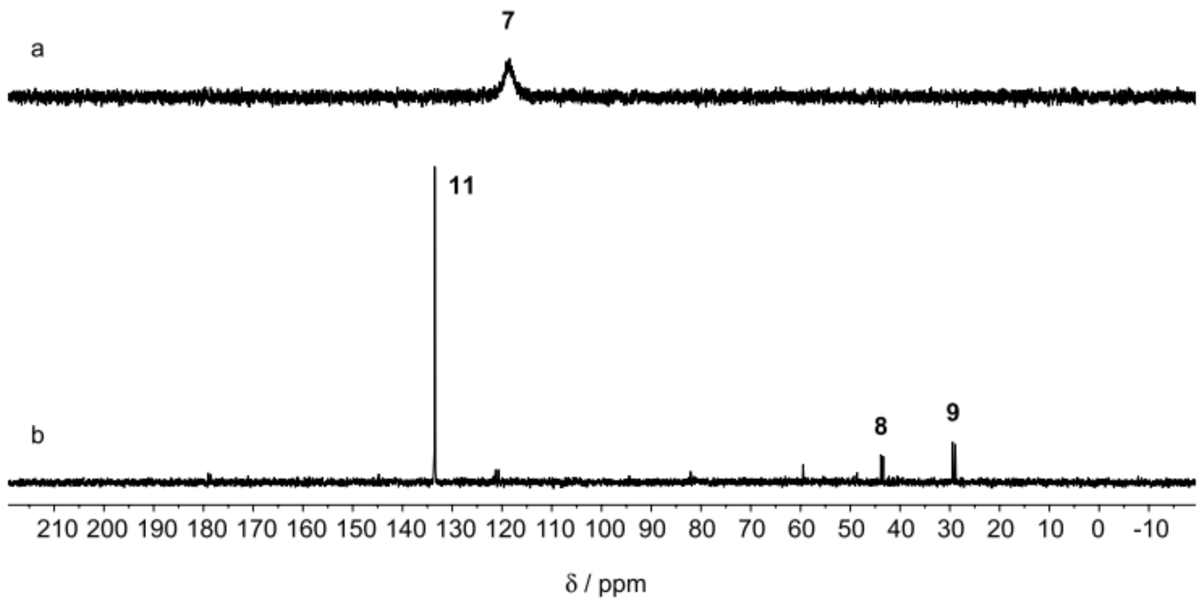

Fig. S7. ^{13}C-NMR Spectra of the reaction mixture with 50 mM ^{13}C-labelled HCN **7** (from $K^{13}CN$), 50 mM NaHS in degassed water (pH = 8, in 10% D_2O in H_2O) (a) before irradiation; (b) after irradiation at 254 nm for 1 hour.

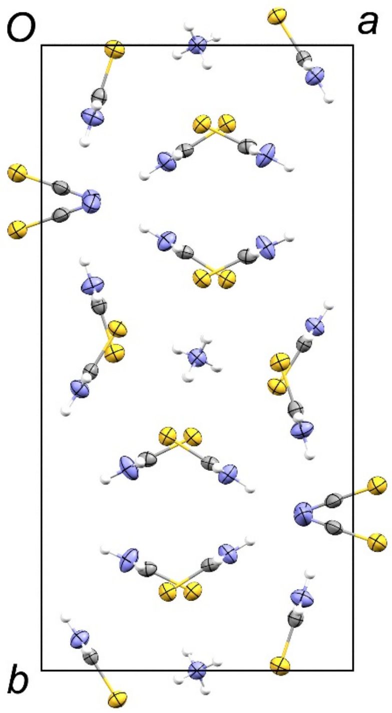

Fig. S8. Crystal structure of the thiourea/thiocyanate inclusion complex (Key: S yellow, N blue, C grey, H white).

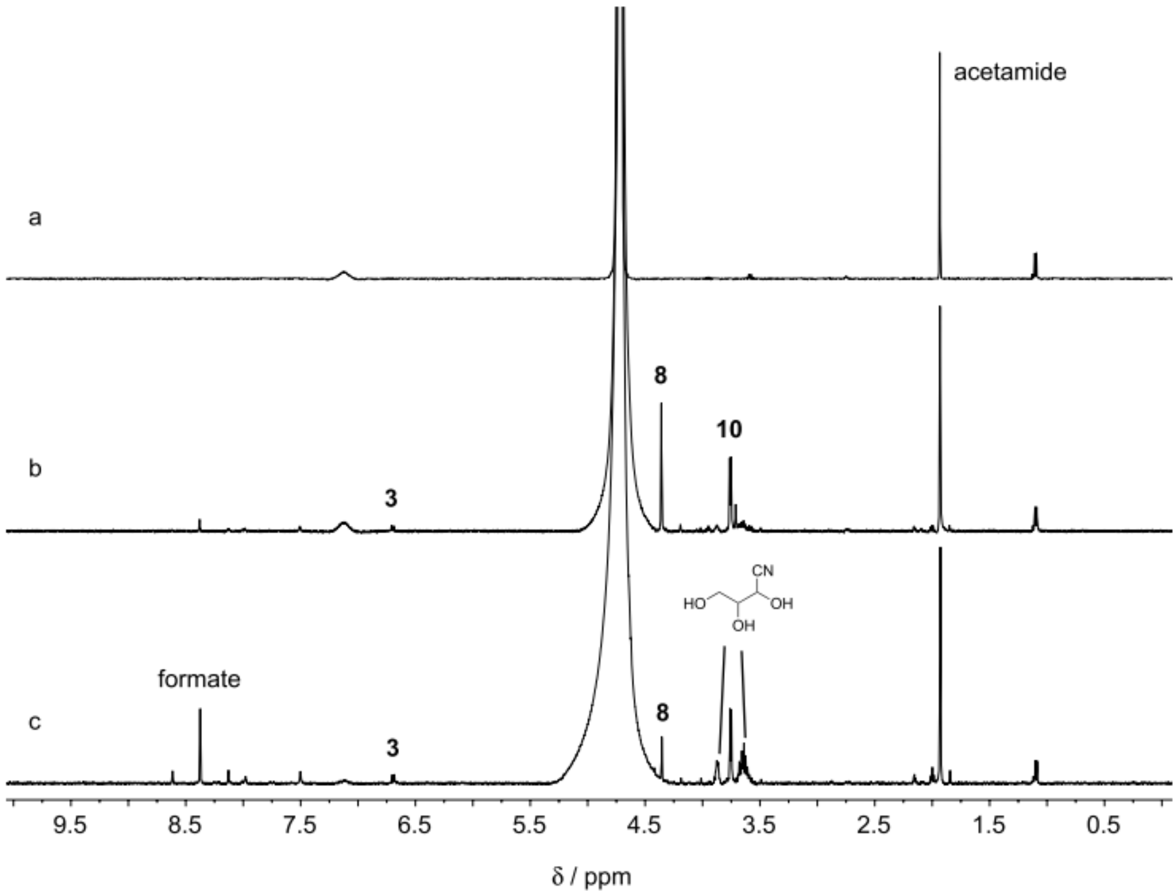

Fig. S9. ^{1}H-NMR Spectra of the reaction mixture with 20 mM ammonium thiocyanate **11**, 80 mM ^{13}C-labelled thiourea **5**, 5 mM potassium ferrocyanide, and 100 mM KCN **7** in 200 mM phosphate buffer (pH = 6, in 10% D_2O in H_2O) (a) before irradiation; (b) irradiation at 254 nm for 19 hours; (c) irradiation at 254 nm for 46 hours.

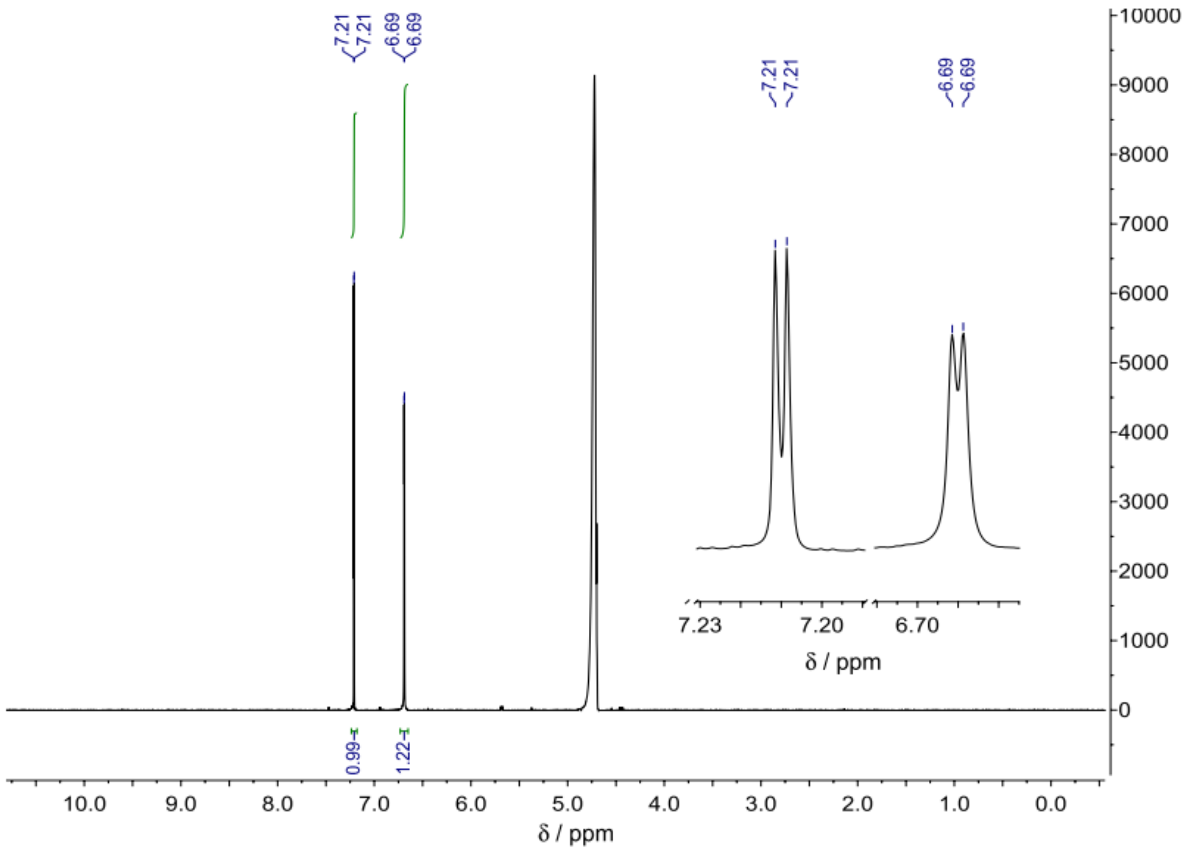

Fig. S10. ^{1}H-NMR spectrum of a standard sample of 2-AO **2**.

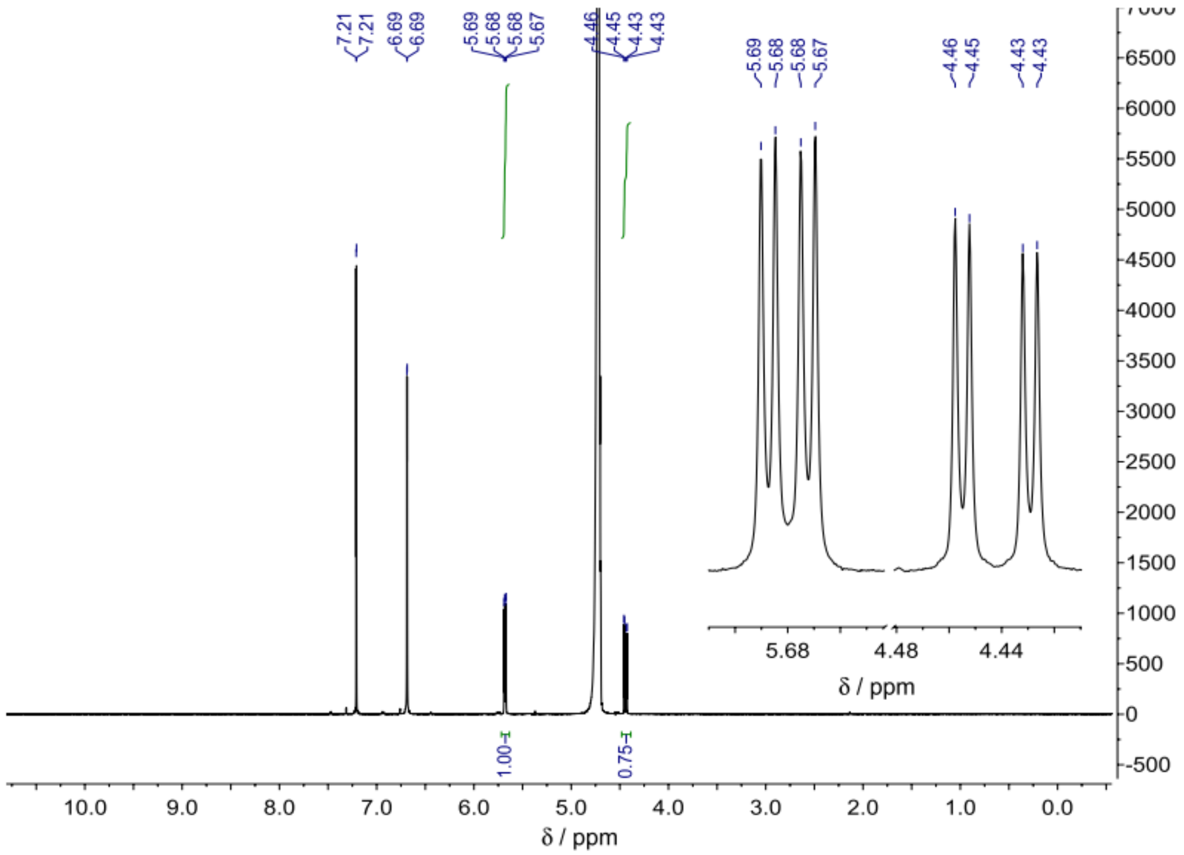

Fig. S11. ^{1}H-NMR spectrum of 2-AO **2** and its hydrate **6**. The missing resonance is obscured by the HOD peak.

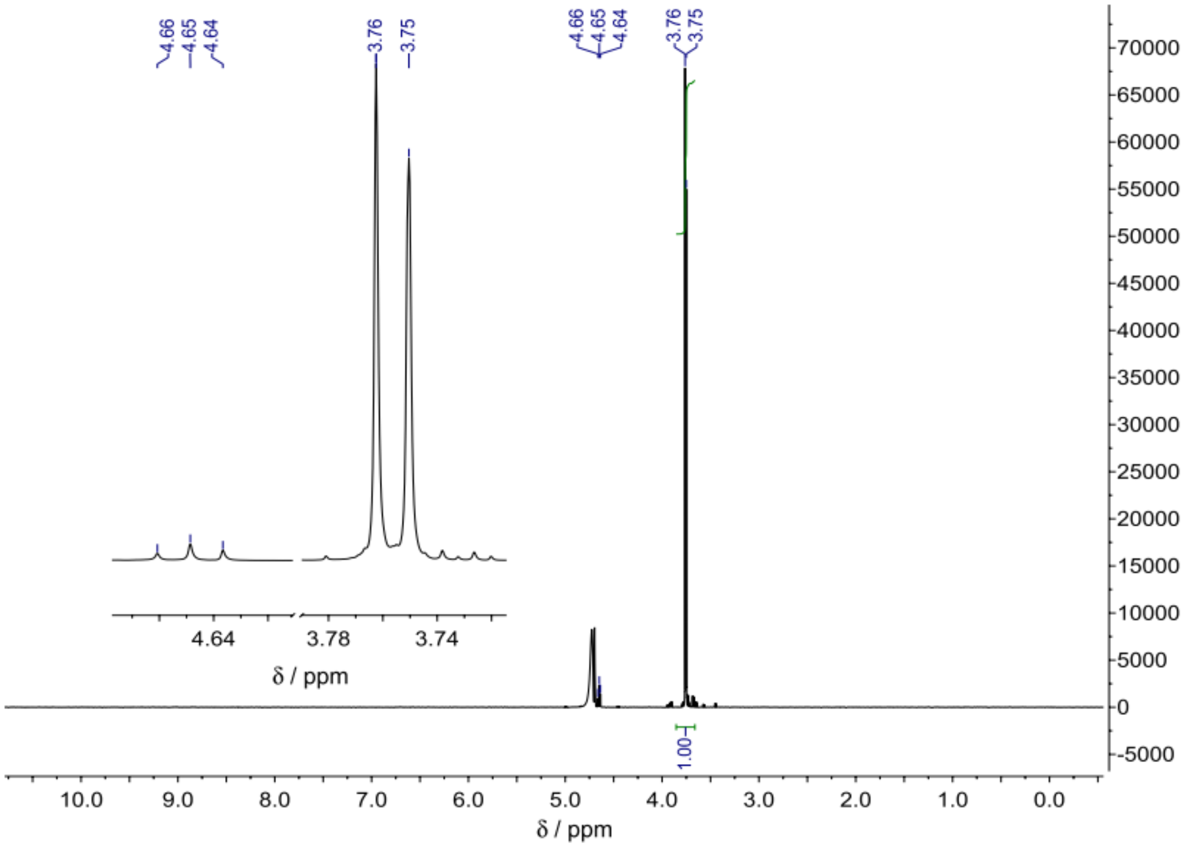

Fig. S12. ^{1}H-NMR spectrum of glyceronitrile **10**.

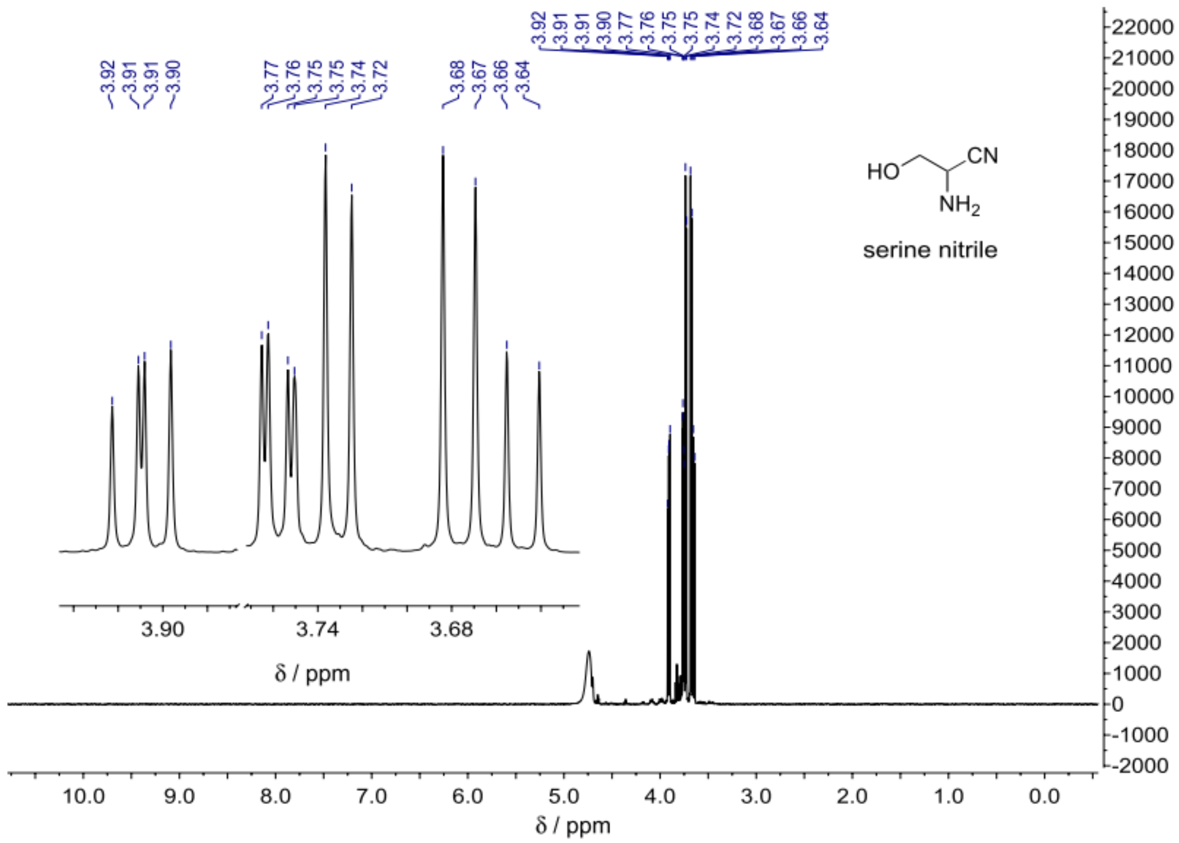

Fig. S13. ^{1}H-NMR spectrum of synthetic of serine nitrile.

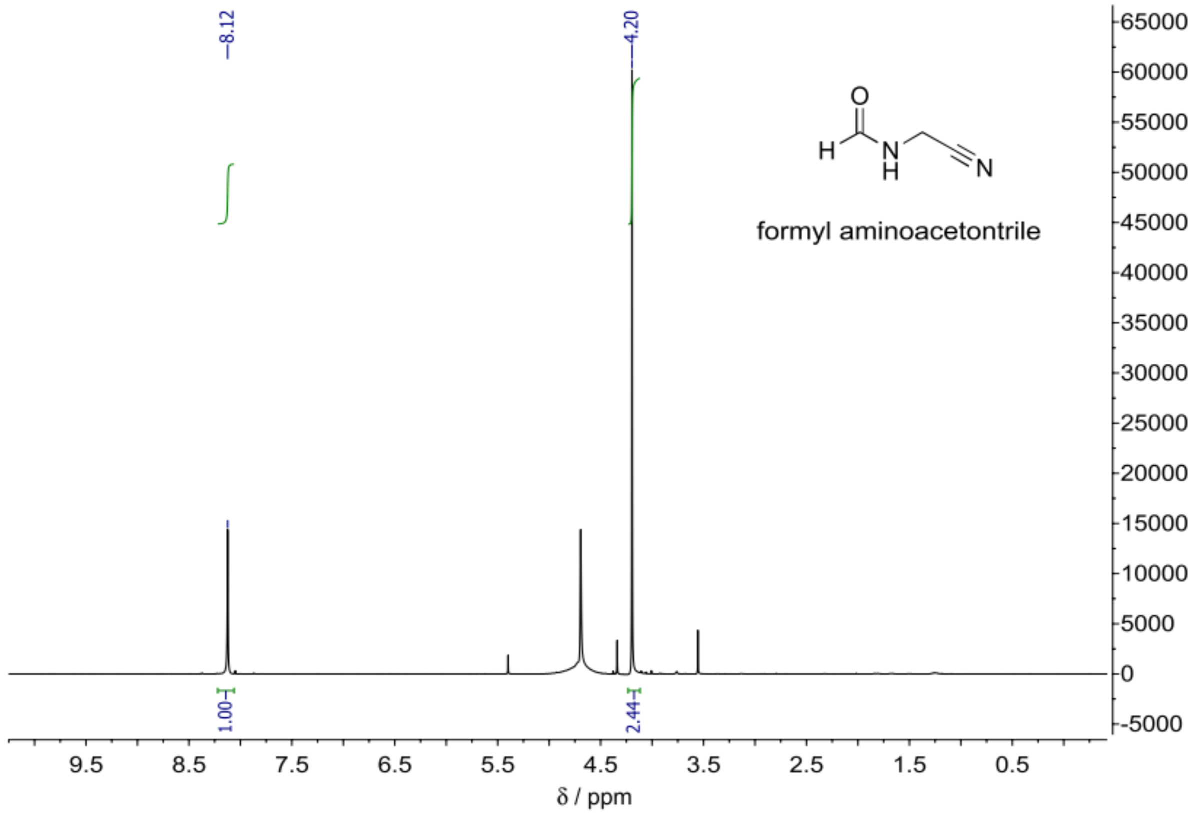

Fig. S14. ^{1}H-NMR spectrum of formyl aminoacetonitrile.

Table S1. Yields of photoredox products in different systems.

Entry	Substrates	pH	Irradiation Time (h)	Products
1	Glycolonitrile (50 mM)	8	7	2-AO (4 %)
	Thiourea (50 mM)			Aminoacetonitrile (8 %)
	Ferrocyanide (5 mM)			Glyceronitrile (27 %)
	KCN (30 mM)			
2	Thiourea (50 mM)	7	6	2-AI (4 %)
	Ferrocyanide (5 mM)			Aminoacetonitrile (28 %)
	KCN (100 mM)			Glycolonitrile (24 %)
				Glyceronitrile (12 %)
3	Thiourea (50 mM)	8	6	2-AO (4 %)
	Ferrocyanide (5 mM)			Aminoacetonitrile (24 %)
	KCN (100 mM)			Glycolonitrile (16 %)
				Glyceronitrile (11 %)
4	Aminoacetonitrile (50 mM)	7	14	2-AI (50 %)
	Thiourea (50 mM)			
	Ferrocyanide (5 mM)			
	KCN (30 mM)			
5	Aminoacetonitrile (50 mM)	8	14	2-AI (20 %)
	Thiourea (50 mM)			
	Ferrocyanide (5 mM)			
	KCN (30 mM)			

Table S2. Crystal and refinement data for the ammonium thiocyanate/thiourea inclusion complex

**CCDC Deposition No.**	**2024098**
Cambridge Data No.	ab_k1_0001
Moiety formula	$(CH_4N_2S)_4 \cdot NCS^{-} \cdot NH_4^{+}$
Sum formula	$C_5H_{20}N_{10}S_5$
MW	380.61
Crystal system	monoclinic
Space group (IT no.)	$P2_1/c$ (no. 14)
a [Å]	10.5332 (5)
b [Å]	21.0945 (8)
c [Å]	8.1916 (3)
α [°]	90
β [°]	94.7245 (13)
γ [°]	90
Z	4
V [Å^3]	1813.93 (13)
D_x [g cm^{-3}]	1.394
Crystal size [mm]	0.22 x 0.08 x 0.05
Crystal colour, shape	colourless block
μ [mm^{-1}]	0.645
T_{min}, T_{max}	0.871, 0.968
Measured reflections	14100
Independent reflections (R_{int})	3171 (0.064)
Observed reflections [I>2σ(I)]	1679
No. of parameters / restraints	193 / 10
$R1$ (observed data)	0.032
$wR2(F^2)$ for all data	0.065
GOF	0.81
Residual electron density [e·Å^{-3}]	–0.30, 0.20
